# Value of MR arthrography for evaluation of children and adolescents with clinically suspected intraarticular cause of hip pain

**DOI:** 10.1007/s00256-023-04552-9

**Published:** 2024-01-11

**Authors:** Laura Schmitt, Christian W. A. Pfirrmann, Florian M. Buck, Thomas F. Hany, Andrea B. Rosskopf

**Affiliations:** 1 Faculty of Medicine, University of Zurich, Zurich, Switzerland; 2 Medical Radiological Institute (MRI) Zurich, Schulthess Clinic, Lengghalde 2, CH-8008 Zurich, Switzerland

**Keywords:** Hip, MRI, Intraarticular contrast, Children, Apophysitis, Labral tears

## Abstract

**Purpose:**

To evaluate the distribution of intra- and extraarticular MRI findings in children and adolescents with clinically suspected intraarticular cause of hip pain in order to assess the need for additional intraarticular contrast administration.

**Material and methods:**

Database was searched over a period of 34 months retrospectively for consecutive hip MR arthrography in young patients (8–17 years) with suspected intraarticular cause of hip or groin pain. Exclusion criteria were prior hip surgery, follow-up examination due to known intraarticular pathology, incomplete examination, qualitatively non-diagnostic examinations, and missing informed consent. Reports of fellowship-trained MSK radiologists were searched for intraarticular versus extraarticular findings explaining hip or groin pain.

**Results:**

Seventy patients (68% female; median age: 14.5 years; range:10.8–16.9 years) were analyzed. No reason for pain was found in 30 (42.9%) hips, extraarticular reasons in 20 (28.6%) cases, intraarticular in 14 (20.0%), and both (intra- and extraarticular) in 6 (8.6%) hips. Most common extraarticular reasons were apophysitis (14.3%), other bony stress reactions (12.9%), intramuscular edema (7%), tendinitis (5.7%), and trochanteric bursitis (4.3%). Labral pathology was the most common intraarticular finding (overall:34.3%; partial tear:15.7%, complete tear:15.7%), most frequent at the anterosuperior position (81.8%). Cartilage defects (1.4%), intraarticular neoplasia (1.4%), and tear of the femoral head ligament (2.8%) were rarely found. Synovitis and loose bodies were not observed. Cam-(37.1%) and pincer-configurations (47.1%) were common while hip dysplasia was rare (5.7%).

**Conclusion:**

MRI in children and adolescents with hip pain should be done primarily without intraarticular contrast administration since most cases show an extraarticular pain reason or no diagnosis detectable with MRI.

## Introduction

Hip pain in children and adolescents is a common cause for pediatric and orthopedic consultations with an overall incidence of 148 cases per 100 000 persons per year [1, 2] and a broad range of infectious, inflammatory, traumatic, developmental, or neoplastic differential diagnosis [3]. The use of hip arthroscopy in pediatric patients has also grown considerably in the recent years [4]. Conventional radiography of the affected side followed by ultrasound is typically recommended as primary imaging tool for diagnostic workup of hip pain in younger children (0–10 years) [1–3]. Common causes of hip pain in this very young age group include transient synovitis, Toddler’s fracture, septic arthritis, osteomyelitis, or Perthes disease [2], and MR imaging is reserved for unclear cases of hip pain. In older children (> 10 years), MRI plays a more important role, since extra- and intraarticular sports injuries like bony stress reactions or labral tears and inflammatory diseases become more frequent with aging [2, 5, 6]. CT imaging is rarely performed in the diagnostic workup due to ionizing radiation. Application of intraarticular contrast media for the MR scan (MR arthrography) can increase the sensitivity for the detection of labral tears and chondral defects and is therefore typically performed in adults with hip pain [5, 7]. A benefit of intraarticular contrast media has also been shown for the preoperative evaluation in children with avascular femoral head necrosis and dysplasia [8–10]. In recent years, we observed an increase of referrals for hip MR arthrography in children and adolescents with hip and groin pain. To date, there is no published study investigating whether contrast administration is also useful in this young age group, especially since arthrography can also cause negative side effects such as pain or infection [11, 12]. Our hypothesis was, that an additional intraarticular contrast application might be unnecessary since most causes of hip pain remain extraarticular in children and adolescents. Therefore, the purpose of our study was to evaluate the distribution (intra- versus extraarticular) of MR arthrography findings in children and adolescents with clinically suspected intraarticular cause of hip pain.

## Materials and methods

This retrospective study was approved by the local ethics committee (Basec-No. 2022-01447). Our institutional PACS system was searched between January 2020 and October 2022 for consecutive patients with the following inclusion criteria: age between 8 and 17 years, suspected intraarticular reason of hip pain, and unilateral MR arthrography of the hip. Exclusion criteria were as follows: prior hip surgery, follow-up examination due to known intraarticular pathology, incomplete examination, qualitatively non-diagnostic examinations and missing informed consent for research purposes.

## MR examination

All patients were scanned in 1.5 or 3 T scanners (Sola/Avanto/Aera or Vida/Skyra; Siemens Healthcare, Erlangen Germany) at the Medical Radiological Institute (MRI) Zurich with the institutional routine hip arthrography MR protocol, an example for the Vida scanner can be found in Table 1. The parameters for the protocols on the other scanners were slightly adapted according to scanner characteristics.

**Table 1:** Routine protocol for 3 T-MR arthrography of the hip in our institution

Sequence	FoV (mm)	Slice thickness (mm)	TE (ms)	TR (ms)
Coronal PDfs	180	3	35	1900
Coronal T1	180	3	11	640
Sagittal PDfs	180	2	35	5000
Axial oblique DESS 3D + radial reconstructions around femoral neck	190	0.8	4.92	11.28
Coronal or axial STIR	380	3.5	40	4720
Femoral torsion HASTE sequence (hips & knees)	380	4	106	1400

*FoV*, field of view; *TE*, echo time; *TR*, repetition time; *ms*, milliseconds; *PDfs*, proton density fat saturated; *DESS*, double-echo steady-state; *STIR*, short tau inversion recovery; *HASTE*, half fourier single-shot turbo spin-echo

## Study readout

All hip MR reports were done by one out of seven fellowship-trained MSK radiologists (experience between 7 and 23 years) in our institution. The reports were screened by a medical student (L.S.), who was trained in report interpretation prior to the study readout by a senior fellowship-trained MSK radiologist (A.B.R.). In unclear cases, the senior MSK radiologist (A.B.R., not involved in the primary report) was consulted by the student for the final diagnosis.

Each report was examined for the following parameters:

### Extraarticular

- Tendinopathy: sprain/tendinitis, partial tear, complete tear
- Muscle pathology: edema, sprain, atrophy, fatty infiltration
- Apophysis: normal or abnormal (bone marrow edema ± fracture)
- Bone pathology: bone marrow edema/stress reaction versus fracture
- Extraarticular tumor
- Bursitis: yes/no (location)
- Other soft tissues (subcutis etc.): edema or other pathology

### Intraarticular

- Labral pathology: signal alteration (no tear), partial tears, or complete tears
- Acetabular cartilage: intact, superficial defect (ICRS (International Cartilage Repair Society) grades 1 and 2), or deep defect (ICRS grades 3 and 4)
- Femoral head cartilage: intact, superficial defect (ICRS grades 1 and 2), or deep defect (ICRS grades 3 and 4)
- Synovitis: yes/no
- Intraarticular tumor: yes/no
- Loose body: yes/no
- Femoral head ligament: intact, partial tear or complete tear
- Supraacetabular fossa: yes/no
- In cases with labral or cartilage pathology, the exact location in the joint was noted: superior, anterosuperior, posterosuperior, anterior, posterior, anteroinferior, posteroinferior, inferior.

The following intraarticular parameters, that do not benefit from contrast application, were rated as separate category (others):

- Cam-configuration: eccentric contour of the femoral head
- Pincer configuration: acetabular retroversion, coxa profunda or protrusio acetabuli
- Hip dysplasia: decreased lateral acetabular coverage
- Osteophytes: yes/no
- Femoral torsion: degrees, antetorsion or retrotorsion

### Summary statement

Potential pain reason: intraarticular, extraarticular, intra- and extraarticular, or no pain reason visible.

### Statistics

Study data were collected and managed using REDCap electronic data capture tools (Version 13.5.4; Vanderbilt University, USA) hosted at the Schulthess Clinic (Zurich, Switzerland) [13]. Descriptive statistics were created using the “Stats & Charts”-Tool in REDCap.

## Results

**Fig. 1:**
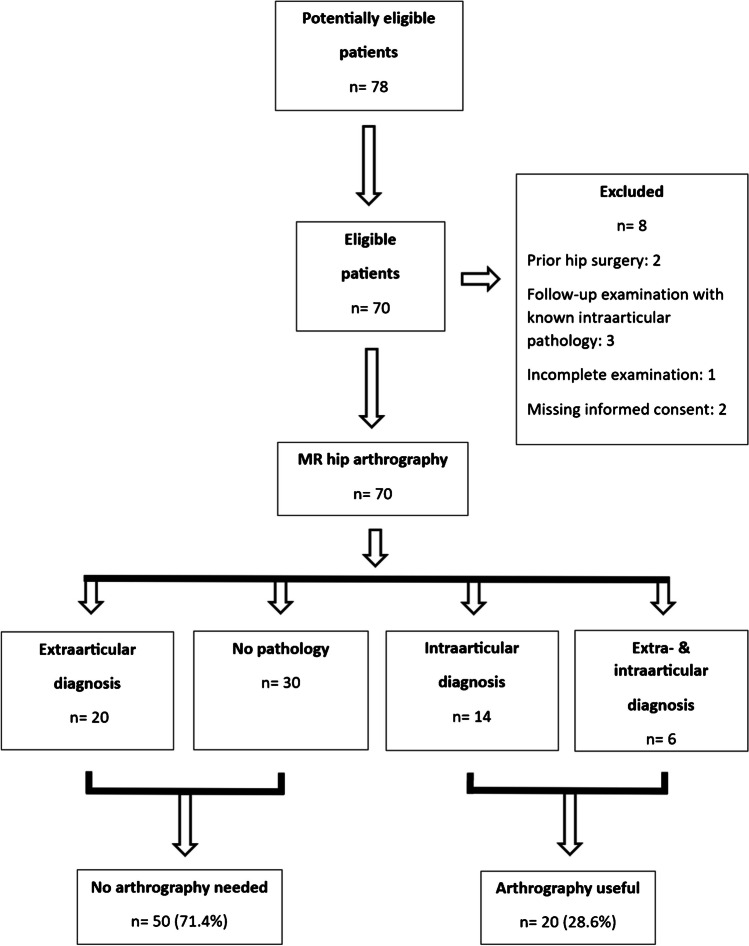
Flow of participants through the study (based on STARD diagram). Diagnosis = possible pain reason

Database search found 78 potentially eligible patients meeting the inclusion criteria. Two patients were excluded due to prior hip surgery, three due to follow-up examination with known intraarticular pathology, one due to incomplete examination, and two to missing informed consent for research purposes (Fig. 1).

In total, 70 patients (68% female) with unilateral MR hip arthrography were included in the analysis with a median age of 14.5 years (range 10.8–16.9 years). Female patients showed a median age of 14.5 years (range 12.4–16.9 years), male patients of 14.5 years (range 10.8–16.0 years). In 57.7% of hips, the right side was examined; in 40.3%, the left side.

No reason for pain was found in 30 (42.9%) cases, extraarticular reasons in 20 (28.6%) hips, intraarticular pathology in 14 (20.0%) cases, and both (intra- and extraarticular reasons) in 6 (8.6%) hips. Therefore, intraarticular contrast application (arthrogram) was not necessary for diagnosis in 71.4% of cases (Fig. 1). Detailed distribution of findings can be seen in Table 2 and 3.

**Table 2:** Summary of findings

	Variables	All (%)	Female (%)	Male (%)
	Total	70/70 (100.0)	49/49 (100.0)	21/21 (100.0)
	Age (mean yrs ± SD; range)	14.5 ± 1.1 (11–17)	14.5 ± 1.1 (12–17)	14.4 ± 1.1 (11–16)
	Side (right/left)	39(55.7)/31(44.3)	25(51.0)/24(49.0)	14(66.7)/7(33.3)
Extraarticular	Bursitis	3 (4.3)	3 (6.1)	0 (0.0)
	Tendons			
	Normal	66 (94.3)	45 (91.8)	21 (100.0)
	Sprain/tendinitis	4 (5.7)	4 (8.2)	0 (0.0)
	Partial tear	0 (0.0)	0 (0.0)	0 (0.0)
	Complete tear	0 (0.0)	0 (0.0)	0 (0.0)
	Muscles			
	Normal	63 (90.0)	42 (85.7)	21 (100.0)
	Edema	7 (10)	7 (14.3)	0 (0.0)
	Sprain	0 (0.0)	0 (0.0)	0 (0.0)
	Atrophy	0 (0.0)	0 (0.0)	0 (0.0)
	Fatty infiltration	0 (0.0)	0 (0.0)	0 (0.0)
	Apophysis			
	Normal	60 (85.7)	45 (91.8)	15 (71.4)
	Bone marrow edema	8 (11.4)	3 (6.1)	5 (23.8)
	Fracture	2 (2.9)	1 (2.0)	1 (4.8)
	Bones			
	Normal	61 (87.1)	41 (85.7)	19 (90.5)
	Bone marrow edema	8 (11.4)	6 (12.2)	2 (9.5)
	Fracture/other pathology	1 (1.4)	1 (2.0)	0 (0.0)
	Neoplasia	1 (1.4)	1 (2.0)	0 (0.0)
	Edema subcutis	3 (4.3)	3 (6.1)	0 (0.0)
Intraarticular	Labrum			
	Normal	46 (65.7)	35 (71.4)	11 (52.4)
	Signal alteration (no tear)	7 (10)	4 (8.1)	3 (14.2)
	Partial tear	11 (15.7)	7 (14.3)	4 (19.0)
	Complete tear	11 (15.7)	6 (12.2)	5 (23.8)
	Cartilage			
	Intact	69 (98.6)	49 (100.0)	20 (95.2)
	Superficial defect	0 (0.0)	0 (0.0	0 (0.0)
	Deep defect	1 (1.4)	0 (0.0)	1 (4.8)
	Femoral head ligament			
	Intact	68 (97.1)	47 (96.0)	21 (100.0)
	Partial tear	1 (1.4)	1 (2.0)	0 (0.0)
	Complete tear	1 (1.4)	1 (2.0)	0 (0.0)
	Synovitis	0 (0.0)	0 (0.0)	0 (0.0)
	Neoplasia	0 (0.0)	0 (00)	0 (0.0)
	Loose bodies	0 (0.0)	0 (0.0)	0 (0.0)
	Supraacetabular fossa	13 (18.6)	11 (22.4)	2 (9.5)
Others	Cam configuration	26 (37.1)	12 (24.5)	14 (66.7)
	Pincer configuration	33 (47.1)	20 (40.8)	13 (61.9)
	Acetabular retroversion	29 (41.1)	16 (32.7)	13 (61.9)
	Coxa profunda	11 (15.7)	10 (20.4)	1 (4.8)
	Protrusion acetabuli	0 (0.0)	0 (0.0)	0 (0.0)
	Hip dysplasia	5 (7.1)	4 (8.2)	1 (4.8)
	Osteophytes	0 (0.0)	0 (0.0)	0 (0.0)
	Femoral torsion*	68 (97.1)	47 (95.9)	21 (100.0)
	Right /left (mean ± SD)	15 ± 11/15 ± 10	19 ± 10/14 ± 11	9 ± 8/18 ± 4
	Antetorsion	58 (82.9)	40 (81.6)	18 (85.7)
	Decreased/retrotorsion	12 (17.1)	9 (18.4)	3 (14.3)

^*^Femoral torsion was assessed in 68 hips; in 2 hips, the sequence for femoral torsion measurement was missing

**Table 3:** Extraarticular and intraarticular diagnosis (potential pain reason)

	All *n* (%)	Female *n* (%)	Male *n* (%)
Total number of patients	70/70 (100.0)	49/49 (100.0)	21/21 (100.0)
Extraarticular	20 (28.6)	14 (28.6)	6 (28.6)
Intraarticular	14 (20.0)	9 (18.4)	5 (23.8)
Extra- AND intraarticular	6 (8.6)	5 (10.2)	1 (4.8)
Unclear / no clear pathology	30 (42.8)	21 (42.8)	9 (42.8)

## Extraarticular findings

**Fig. 2:**
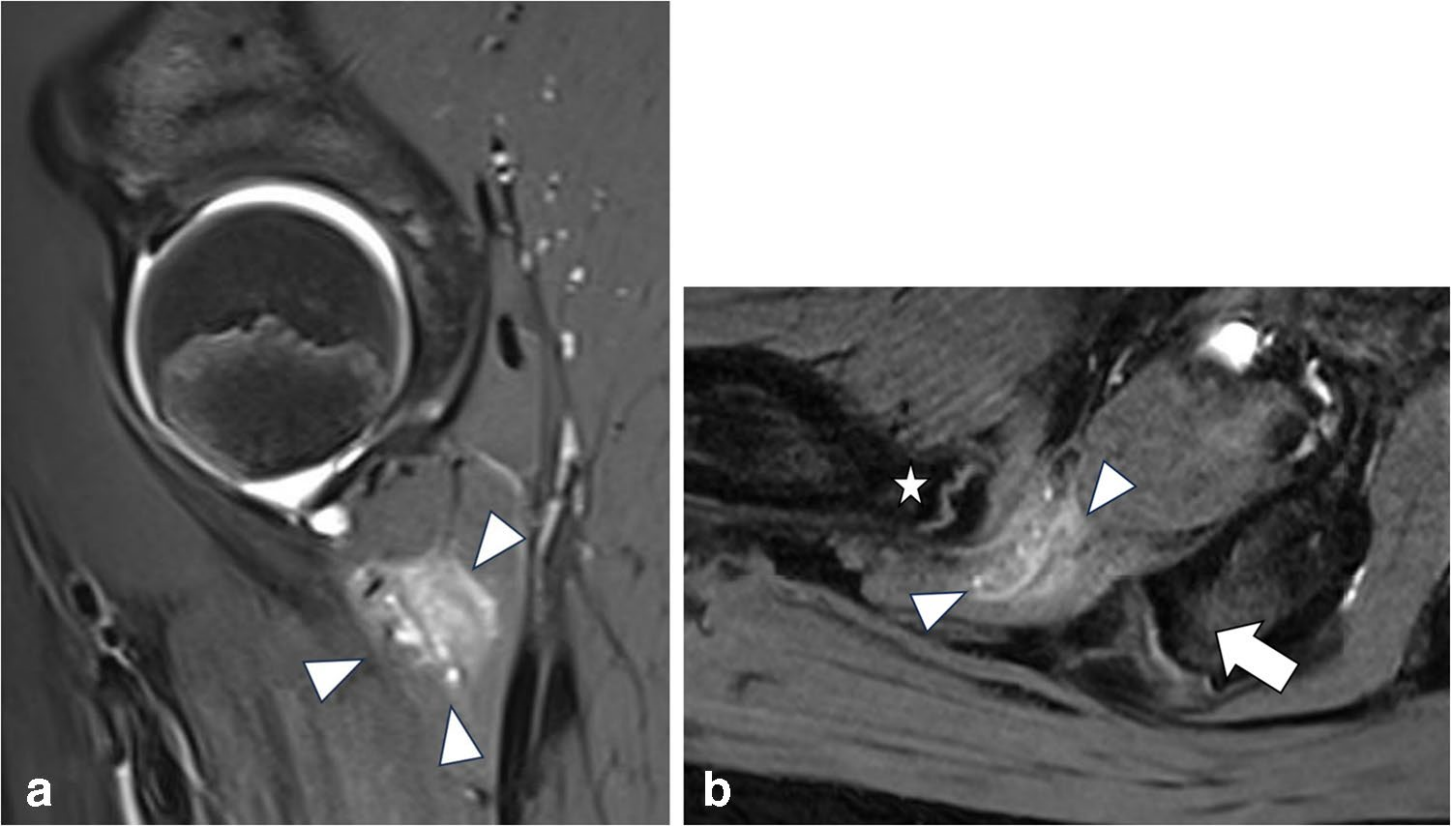
**A** Sagittal PDfs sequence in a 13-year-old girl shows edema in the right quadratus femoris muscle. **B** Axial oblique DESS-sequence in the same patient showing edema (arrowheads) in the ischiofemoral space between between lesser trochanter (asterisk) and lateral cortex of the ischial tuberosity (arrow). The patient also showed a decreased distance (9 mm) between lesser trochanter and lateral cortex of the ischial tuberosity on axial HASTE torsion sequence (not shown), suggesting the diagnosis of ischiofemoral impingement syndrome

**Fig. 3:**
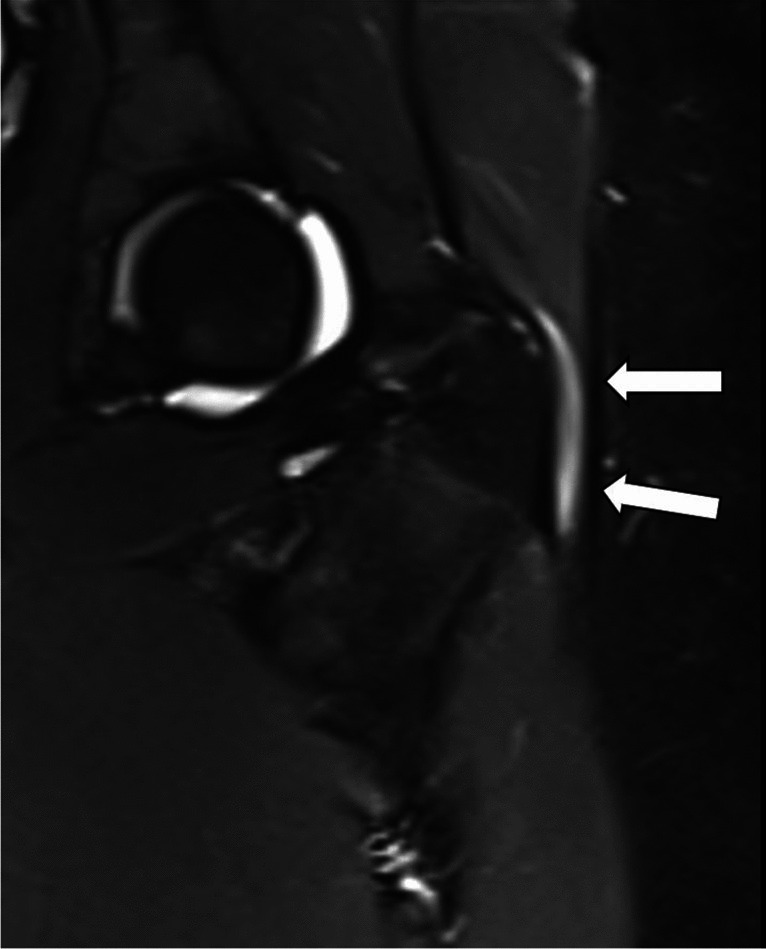
Coronal STIR sequence in a 14-year-old girl with trochanteric bursitis (arrows)

**Fig. 4:**
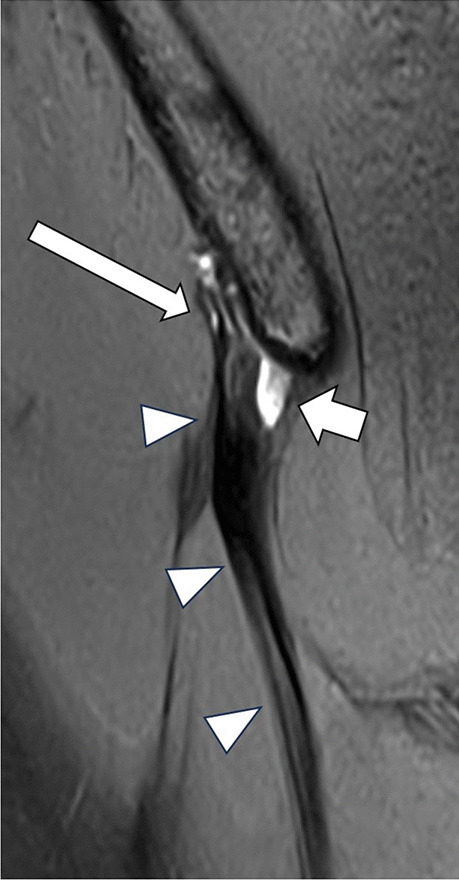
Fifteen-year-old girl with edema (long arrow) surrounding the origin of the rectus femoris tendon (arrowheads) and ganglion cyst formation (short arrow) on coronal PDfs sequence

**Fig. 5:**
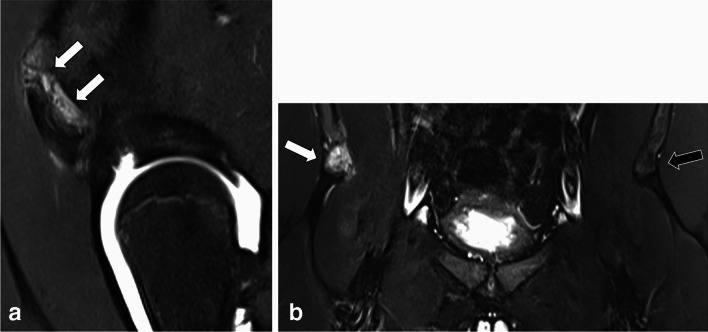
**A** and **B** Fourteen-year-old boy with acute avulsion of the apophysis (arrow) of the right anterior inferior iliac spine on sagittal PDfs sequence (**A**). **B** shows coronal STIR sequence of the same patient with apophyseal avulsion on the right side (white arrow) and the normal apophysis on the left side (black arrow)

**Fig. 6:**
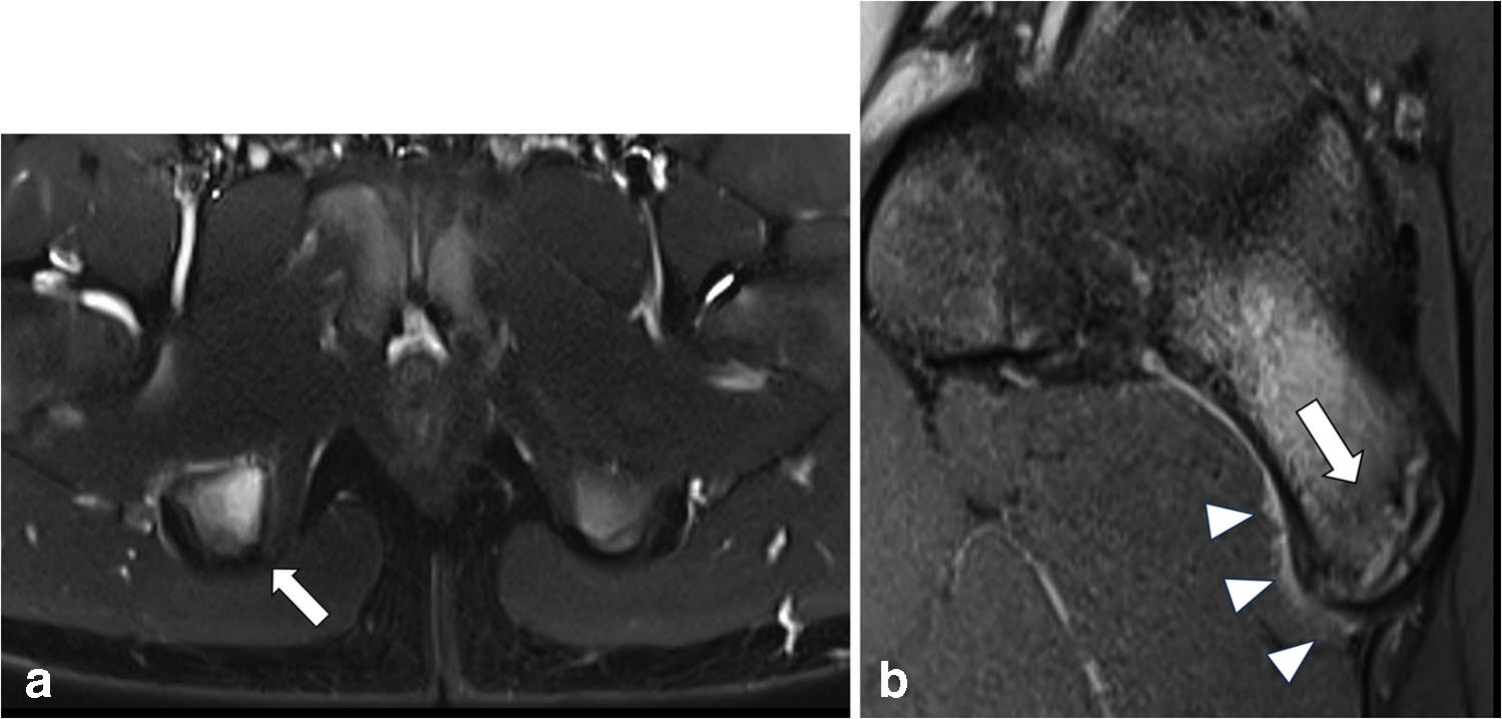
**A** Fourteen-year-old boy with stress reaction in the right ischial tuberosity at the origin of the hamstring tendons on axial STIR sequence. **B** Corresponding sagittal pdfs sequence shows edema in the ischial tuberosity (arrow) and adjacent periostitis with soft tissue edema (arrowheads)

**Fig. 7:**
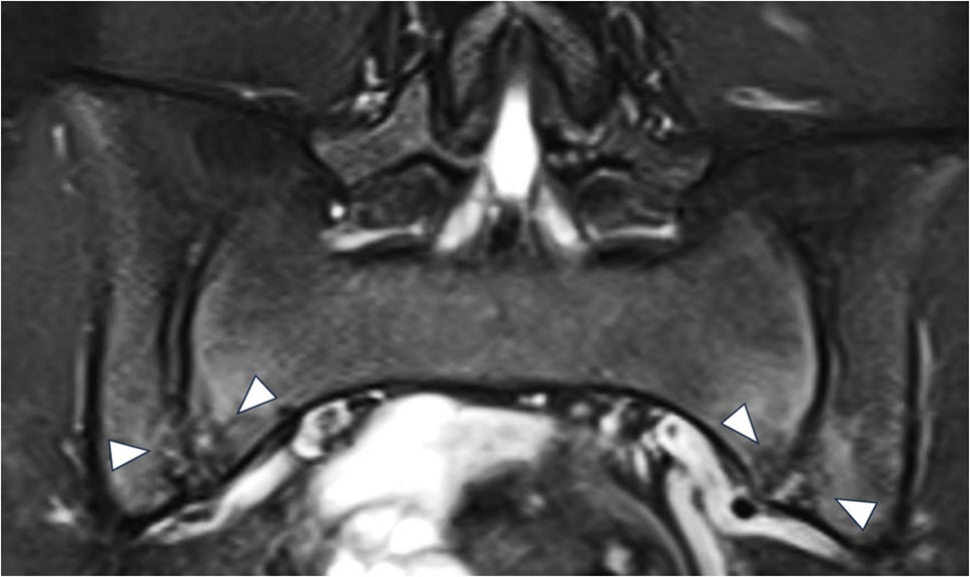
Fifteen-year-old boy with incidental finding of bilateral cortical irregularities and bone marrow edema (arrows) at the inferior parts of the sacroiliacal joint (SIJ) on coronal STIR sequence (A) of the routine MR hip arthro protocol. The patient was later on diagnosed with juvenile spondyloarthritis

In seven girls muscle edema was detected, in six of these cases in the quadratus femoris muscle (Fig. 2A, B), and in one case in the gluteus medius muscle. Bursitis was seen in three girls in the trochanteric bursa (Fig. 3). No iliopectineal bursitis was found. Isolated tendon sprain/tendinitis was seen in only four cases, in two of them at the insertion of the gluteus medius tendon at the greater trochanter, in one case at the origin of the hamstring tendons and in one patient in the proximal rectus femoris tendon (Fig. 4). Partial or complete tendon tears were not detected. Apophyseal avulsion injury (fracture) was seen in two boys at the anterior inferior iliac spine (example Fig. 5A, B). Apophyseal edema was seen in eight patients, in four of them in the anterior inferior iliac spine, two of them in the anterior superior iliac spine, and two of them in the ischial tuberosity (example Fig. 6A, B). Other bone marrow edema was seen in eight patients: in the ischial tuberosity, the anterior inferior iliac spine (with fused apophysis), in the proximal femur, the iliac scoop and the symphysis pubica. On case also showed edema of the sacroiliac joints (Fig. 7). The only case with extraarticular neoplasia was an osteochondroma at the femoral neck with intraarticular extension.

## Intraarticular findings

**Fig. 8:**
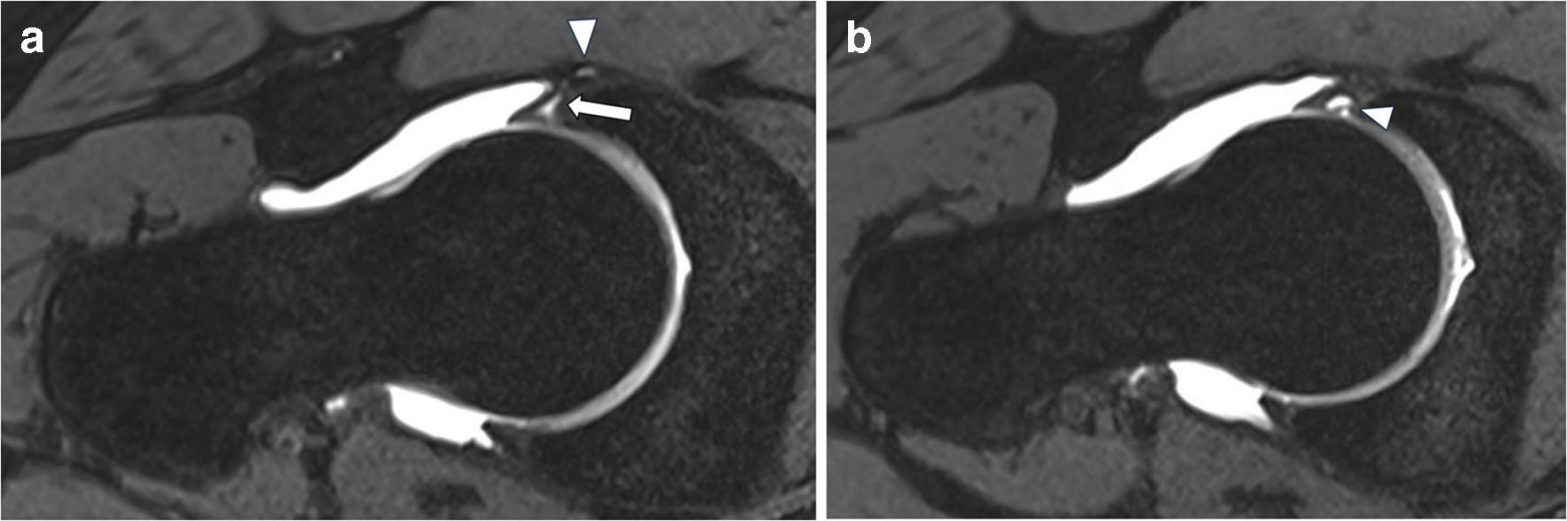
**A** Fourteen-year-old girl with complete tear (arrow) at the anterosuperior labral base with adjacent paralabral ganglion cyst formation (arrowhead) on oblique axial DESS sequence. **B** Oblique axial DESS sequence (slightly inferior to Fig. 5A) shows additional intralabral ganglion (arrowhead) in the same patient

**Fig. 9:**
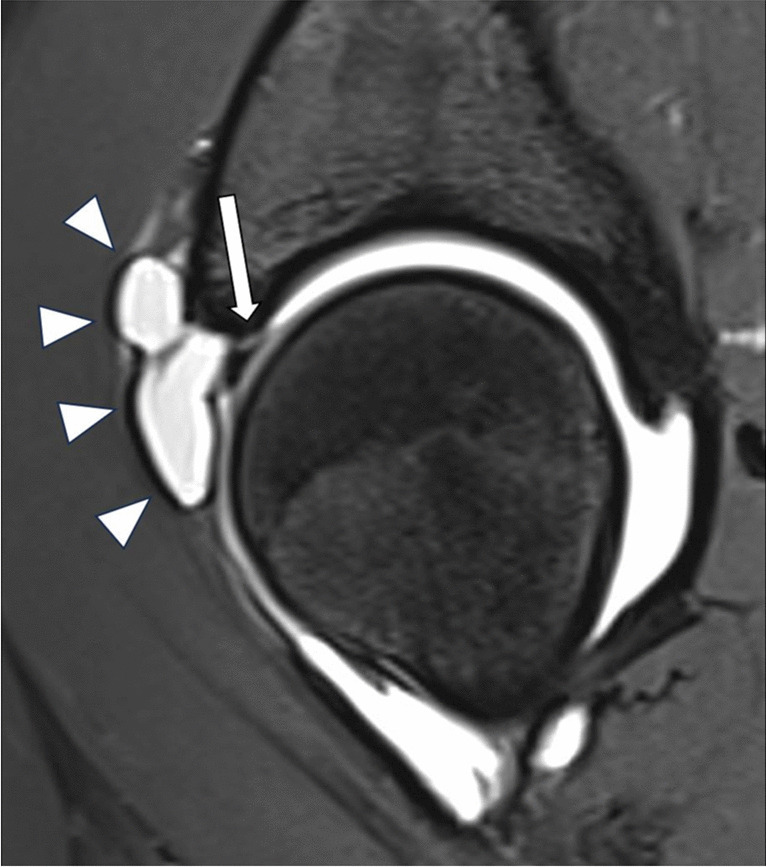
Sagittal PDfs sequence (B) in a 14-year-old girl shows complete tear (arrow) of the anterosuperior labrum of the left hip and adjacent ganglion cyst formation (arrowheads)

Partial or complete labral tears were seen in 22 cases. Most tears were located anterosuperior (81.8%) and anterior (18.2%) at the acetabulum (examples see Figs. 8A, B and 9). Fourteen of the 22 partial or complete labral tears were clearly visible, the others described as probable tearing. Signal alteration of the labrum without tearing was seen in seven cases, all of them at the anterosuperior position.

A single deep cartilage defect was seen at the anterior acetabulum in one male adolescent.

## Femoral torsion

Femoral torsion was assessed in 68 of 70 hips according to the method by Tomczak et al. [14] measuring the angle between a line connecting the center of the femoral head with the center of the femoral neck at its base and the tangent line at the dorsal border of the femoral condyles. Decreased antetorsion was defined as a femoral torsion angle < 5°. Increased antetorsion was defined as femoral torsion > 30°.

Decreased antetorsion or retrotorsion was seen in 17.1% of cases, more often in girls (18.4%) compared to boys (14.3%). The highest antetorsion was 36° in girls and 24° in boys. Four girls had a femoral antetorsion > 30°.

## Discussion

MR arthrography of the hip in children and adolescents with hip pain with a clinically possible intraarticular cause shows more extraarticular than intraarticular pain reasons. Furthermore, in many cases (43%) no pain explanation is found with MR imaging.

Labral tears were the most common type of intraarticular pathology found in this study, in 15.7% with partial tearing, and 15.7% with complete tearing. A recent study by Georgiadis et al. [15] showed that the prevalence of asymptomatic labral tears is very low in children with 1.4% confirming similar findings of an older study by Aydingöz et al. [16]. The latter stated a linear signal intensity in the labrum of asymptomatic adolescents on a fluid sensitive native MR sequence in only 3.6% of cases. For this reason, it is very likely that the labral tears in our study are causing the hip pain. The relative high number of labral tears in our study might be explained by a good preselection of the study cohort: All children had a suspected intraarticular cause of hip pain and were referred by specialized pediatric orthopedic surgeons or pediatricians with expertise in orthopedic examination of children and adolescents. The most common site for labral tearing was the anterosuperior part of the acetabulum, which is consistent with previous studies in adults [17, 18].

Only one patient had a visible cartilage defect. This finding is no surprise, since cartilage defects are in general most likely associated with osteoarthritis, which is a very rare disease under the age of 40. Other rare diseases associated with cartilage defects or loss of cartilage in children and adolescents [19–21], like Perthes disease, chondrolysis of the hip, slipped capital femoral epiphysis, severe trauma, prolonged immobilization, infective arthritis, and juvenile idiopathic arthritis were not seen and in addition, an already known hip pathology was an exclusion criterium. The presence of a supraacetabular fossa in our study (18.6%) was lower compared to 35.6% in the study by Vaeth et al. [22]. Maybe the supraacetabular fossa was not mentioned by all radiologists in their report since this is only a frequent anatomical variant in young patients. It is also known as pseudodefect of the acetabular cartilage and visible as a focal defect in the subchondral bone of the acetabular roof at the 12 o’clock position, partially or completely filled with cartilage [22].

Most common extraarticular pathology in this study were apophyseal stress reactions/avulsion injuries in 14.3% of cases, especially in the apophysis of the inferior iliac spine. This is consistent with the current literature, since these injuries are very common in adolescents [23]. The iliac crest (abdominal muscle insertion), anterior superior iliac spine (sartorius insertion), anterior inferior iliac spine (rectus femoris origin), ischial tuberosity (hamstring origin), and lesser trochanter (iliopsoas insertion) are known as frequent sites of apophyseal injury, similar to our study findings. These injuries might be undetectable on normal radiographs, but are easily visible on MRI [24]. Second most common extraarticular pathology was muscle edema, most often in the quadratus femoris muscle. Edema in the quadratus femoris muscle is frequently associated with ischiofemoral impingement syndrome [25], which may cause anterior or posterior hip pain [26]. This pathology is frequently seen in elderly patients but has also been described in children down to four years of age, often in association with coxa valga formation [27] or torsional abnormalities of the femur. Trochanteric bursitis was rarely seen and only in female patients. This pathology is rarely described in children and adolescent athletes [28], but common in middle-aged women [29] causing lateral hip pain.

Our study results showed some differences between genders. Tendon pathologies, signs of bursitis and also subcutaneous edema were only seen in female, but not in male patients. Furthermore, edema in the quadratus femoris muscle was only visible in female patients. This finding is consistent with previous studies in children and adolescents with about 75% of female patients presenting with ischiofemoral impingement [27, 30, 31]. On the other hand, bone marrow edema was much more frequent in the apophysis of male patients (23.8% versus 6.1%), which could be explained by the age distribution of the patients with a median age of 14.5 years. In boys apophyseal and epiphyseal injuries typically occur at 13 to 14 years, in girls significantly earlier at 11 to 12 years [32]. Cam configuration was much more frequent in boys (66.7%) than in girls (24.5%), which is consistent with the current literature [33].

The typical investigation pathway in children with hip pain includes radiographs as first choice, followed by ultrasound or MRI [2]. MRI is excellent for assessment of soft tissues, cartilage, joints and bone marrow. It has high sensitivity and specificity, and is very useful for confirming osteomyelitis, detection and extent of malignancies, and for identifying subtle stress fractures [2]. The number of normal MRI scans (42.8%) in this study was higher than in previous published studies about children with acute hip pain (Ranner et al.: 16% [6]; White et al.: 12%[5]). However, the previous studies were done more than 20 years ago, where MR scanner were less available and the threshold for MR scanning much higher than in medicine today, which might explain the mismatch of these numbers.

A limitation of this study is the retrospective study design based on the information written on the referral form. The patients’ detailed physical examination data were not available. Therefore, a clear correlation between MR findings and pain correlation could not be done. This was specifically difficult regarding the high number of impingement configurations and femoral torsion abnormalities in this study. An isolated impingement configuration or torsion abnormality is also a known frequent finding in asymptomatic people [34, 35]. Furthermore, other common causes of hip pain in pediatric populations are not identified in the study, such as septic arthritis, transient synovitis, Perthes disease, juvenile rheumatoid arthritis, and slipped capital femoral epiphysis. This could be related to selection bias and diminishes the generalizability of the findings presented. Finally, the results of the study are derived from the review of radiology reports only and not from a study-related systematic radiological review of the images themselves, which might lead to incomplete mentioning of findings due to non-standardized reporting.

In summary, most children and adolescents with a clinically suspected intraarticular cause of hip pain do not benefit from additional intraarticular contrast administration because most MRIs are normal or show extraarticular pathology. Therefore, MR arthrography of the hip should be preserved as second-line examination in children and adolescents for cases with clearly suspected labral or chondral pathology. Because of the high incidence of extraarticular pathology, an additional fluid-sensitive sequence with a large field of view covering the whole pelvis is recommended in these young patients in order to detect extraarticular reasons for hip pain like apophyseal stress reactions or sacroiliitis.

## Data Availability

The data that support the findings of this study are available from the corresponding author (A.B.R.), upon reasonable request.

## Declarations

All procedures performed in studies involving human participants were in accordance with the ethical standards of the institutional and/or national research committee and with the 1964 Helsinki declaration and its later amendments or comparable ethical standards.
